# Genetic associations between human-directed behavior and intraspecific social aggression in growing pigs

**DOI:** 10.1093/jas/skad070

**Published:** 2023-03-04

**Authors:** Suzanne Desire, Julia A Calderón Díaz, Craig R G Lewis, Rainer Roehe, Simon P Turner

**Affiliations:** SRUC (Scotland’s Rural College), West Mains Road, Edinburgh, Scotland EH9 3JG, UK; PIC Europe, C/Pau Vila, 22 2o piso, 08174, Sant Cugat del Valles, Barcelona, Spain; PIC Europe, C/Pau Vila, 22 2o piso, 08174, Sant Cugat del Valles, Barcelona, Spain; SRUC (Scotland’s Rural College), West Mains Road, Edinburgh, Scotland EH9 3JG, UK; SRUC (Scotland’s Rural College), West Mains Road, Edinburgh, Scotland EH9 3JG, UK

**Keywords:** aggression, boldness, fear, human–animal interactions, pigs

## Abstract

This study estimated the genetic parameters for human-directed behavior and intraspecific social aggression traits in growing pigs, and explored the phenotypic correlations among them. Data on 2,413 growing pigs were available. Pigs were mixed into new social groups of 18 animals, at 69 ± 5.2 d of age and skin lesions (**SL**) were counted 24 h (**SL24h**) post-mixing. Individual behavioral responses to isolation in a weighing crate (**CRATE**) or when alone in an arena while a human directly approached them (**IHAT**) were assessed within 48 h post-mixing. Additionally, pigs were tested for behavioral responses to the presence of a single human observer walking in their home pen in a circular motion (**WTP**) within one (**T1**) and 4 wk post-mixing (**T2**) noting pigs that followed, nosed or bit the observer. Animal models were used to estimate genetic and phenotypic parameters for all studied traits. Heritabilities (**h**^**2**^) for SL, CRATE and IHAT responses were low to moderate (0.07 to 0.29), with the highest h^2^ estimated for speed of moving away from the approaching observer. Low but significant h^2^ were estimated for nosing (0.09) and biting (0.11) the observer at T2. Positive high genetic correlations (**r**_**g**_) were observed between CRATE and IHAT responses (0.52 to 0.93), and within SL traits (0.79 to 0.91) while positive low to high correlations between the estimated breeding values (**r**_**EBV**_) were estimated within the WTP test (0.24 to 0.59) traits. Positive moderate r_g_ were observed between CRATE and central and posterior SL24h. The r_EBV_ of CRATE and IHAT test responses and WTP test traits were low, mostly negative (−0.21 to 0.05) and not significant. Low positive r_EBV_ (0.06 to 0.24) were observed between SL and the WTP test traits. Phenotypic correlations between CRATE and IHAT responses and SL or WTP test traits were mostly low and not significant. Under the conditions of this study, h^2^ estimates for all studied traits suggest they could be suitable as a method of phenotyping aggression and fear/boldness for genetic selection purposes. Additionally, genetic correlations between aggression and fear indicators were observed. These findings suggest selection to reduce the accumulation of lesions is likely to make pigs more relaxed in a crate environment, but to alter the engagement with humans in other contexts that depends on the location of the lesions under selection.

## Teaser

Aggression and fear/boldness indicators in pigs are heritable and there is evidence of genetic associations between them. Selecting against extremely shy and extremely bold pigs could result in easier to handle pigs while performing certain routine farm procedures.

## Introduction

In recent years, temperament traits such as aggressiveness or fearfulness have received increasing attention in farming operations, as they affect how the animals respond to different husbandry practices (Haskell et al., 2014; Norris et al., 2014). The increased demand for meat products has led to a rapid growth in the scale and intensification of livestock systems (Azarpajouh et al., 2021). Changes in production systems have resulted in lower stock person per animal ratio and therefore, in less opportunities for animals to become habituated to the presence of and being handled by humans when necessary (Holl et al., 2010; von Borstel et al., 2019). Animals may become more fearful when interacting with stock personnel which could contribute to chronic stress and possibly affect other fundamental behaviors such as social interactions (Forkman et al., 2007). At the same time, re-grouping is a common practice on pig farms (Rodrigues da Costa et al., 2021) leading to agonistic interactions as new dominance relationships need to be established (Fels et al., 2014). Therefore, selection of calmer, easier to handle and less aggressive pigs is vital to improve their ability to adapt to new challenges and reduce stress during routine farming procedures, thereby improving their well-being.

Heritabilities for behaviors thought to measure fearfulness and the ability to cope in stressful situations are low to moderate (D’Eath et al., 2009; Holl et al., 2010; Rohrer et al., 2013; Scheffler et al., 2014) and it is likely that these behaviors are genetically associated with social aggression. For example, D’Eath et al. (2009) reported a genetic correlation of 0.10 ± 0.02 between movement and vocalizations during weighing and aggressive behavior at mixing, suggesting a shared genetic basis between reaction to human presence, social isolation and/or restraint (all components of weighing) and intraspecific aggression. At a phenotypic level, more reactive pigs and pigs that were quicker to touch a novel object while in isolation also performed higher levels of aggression (Ruis et al., 2000; Bolhuis et al., 2005a, 2005b; Melotti et al., 2011). However, before including these traits as selection objectives, a better knowledge of the relationships between aggression and fear responses is required for the effective integration of behavioral traits into new pig breeding programs. Therefore, this study aimed to estimate genetic parameters for human-directed behavior and intraspecific social aggression traits in growing pigs, and to explore the phenotypic correlations among them.

## Materials and Methods

### Ethics approval

The procedures described were approved by the institutional Animal Ethics Committee (ED-AE-43-2012). Governmental licensing was not required.

### Animal management

Data were collected between December 2013 and June 2014 on 2,413 growing pigs (*n* = 1,202 females and *n* = 1,211 barrows [castrated males]) from a commercial sow herd belonging to the Pig Improvement Company (**PIC)** where multiple lines were crossed onto the sows. The farm was located in South Eastern USA. Each pig was individually identified with an ear tag. Pigs were progenies of 116 sires and 391 dams and originated from seven different PIC terminal genetic lines. Pedigree information was available for two generations (i.e., grandparents, *n* = 4,104 animals). Pigs were mixed in single sex groups (*n* = 18 pigs per group) of mixed genetic line at approximately 69 ± 5.2 d of age and they remained in the same groups until the end of the test period. Groups were formed by mixing nine pigs from two non-adjacent weaning pens. Groups that were mixed on the same day were regarded as being in the same batch. Eight groups were formed per batch and 17 batches were used in total to generate a total of 138 groups (batch 1 contained 10 pen groups). On average, animals from 11.6 ± 2.1 litters were represented in each group, and the mean number of pigs per litter per pen was 1.5 ± 0.81 pigs. Animals were housed in pens with fully slatted floors with a minimum space of 0.65 m^2^ per pig. Dry pelleted feed was provided ad libitum and pigs had constant access to water via nipple drinkers.

### Measurements

#### Weigh crate response and individual human approach test

**Figure 1.**
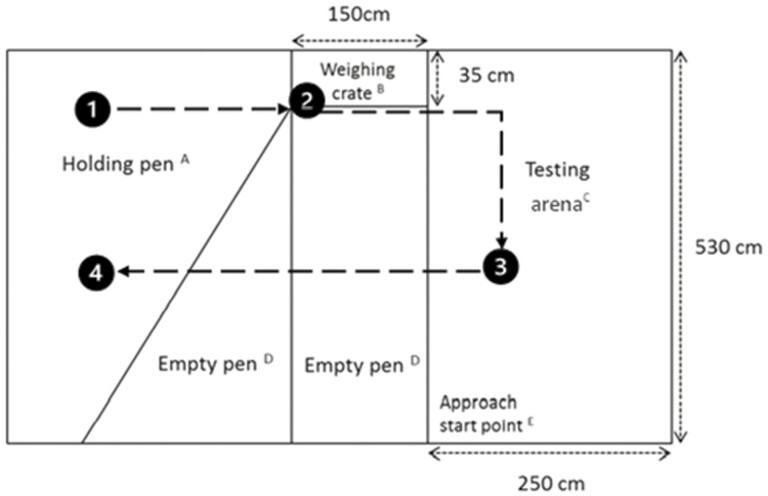
Diagram illustrating the layout of the testing area and testing process for the crate response and individual human approach test. 1) The entire group of pigs were held in the holding pen (A). 2) Each pig was individually moved to the weighing crate (B) and their behavioral response was recorded. 3) After approximately 1 min each pig was then moved to the testing pen (C) and the behavioral response to a human walking toward them from the lower left corner (E) was recorded. 4) Pigs were returned to the holding pen (A) with the rest of the group after testing.

Behavior of individual pigs while isolated was assessed within 48 h post-mixing. All pigs were handled and tested by a single trained observer. Each group of pigs was transferred from their home pen into an experimental arena (Figure 1), where two different behavioral tests were conducted. First, pigs were moved to a holding pen and each pig was individually moved into the weighing crate using a plastic stock board to assess their response to isolation while in the crate (**CRATE**). Pigs remained isolated in the weighing crate for approximately 1 min and they were scored based on their restlessness on a 4-point scale, where 1 = pig performing exploratory behavior including sniffing and rooting of the crate floor and walls; 2 = pig shifting from side to side, attempts to turn; 3 = pig performing vigorous movements, attempts to escape by turning or running backwards and forwards; and 4 = pig performing serious, persistent attempts to escape by jumping over crate wall. Once the crate response test was completed, the pig was released into an empty testing arena and the individual human approach test (**IHAT)** was conducted. Approximately 30 s after the pig entered the testing arena, the observer walked toward the pig at a steady pace starting in the same corner of the arena each time and recorded the pig’s reaction. Three separate scores were given for each pig based on the severity of their movement (**MOVEMENT**), vocalizations (**VOCALISE**), and vigilance (**VIGILANCE**; Table 1).

**Table 1.** Scoring systems used to assess individual behavioral responses in growing pigs isolated in a pen to a human approach within 48 h post-mixing

Score	Movement	Vocalization	Vigilance
0	None	None	None
1	Walk	Quiet grunts	Medium (i.e. occasional glances at human)
2	Trot	Loud grunts/squeals	High (i.e. completely focused on human)
3	Run	-	-

#### Walk-the-pen test

The walk-the-pen (**WTP)** test was designed as a practical approximation of pig-human interactions that occur while a producer performs the daily walk around the pens to ensure appropriate animal care. Pigs were tested for behavioral responses to the presence of a single human observer in their home pen at 6 ± 4.9 (**T1**) and 25 ± 15.9 (**T2**) d post-mixing. To begin the test, the observer entered the pen by climbing over the gate and walked once around the perimeter of the pen at a normal speed to ensure all animals were alert and aware of the human presence. The observer then walked around the pen a second time and recorded the ear tags of each pig that followed the observer for more than 0.5 laps of the pen. At the end of the second lap, the observer paused for 1 min and noted individuals that performed the following behaviors: 1) ***NOSE*** (i.e., nosed or rooted at the observer’s boots or legs); 2) ***FOLLOW*** (i.e., pig followed the observed around the pen), or 3) ***BITE*** (i.e., pig bit at the observer’s legs) the observer.

### Skin lesions

Skin lesions, as a proxy of aggressive interactions, were counted immediately prior to mixing, and 24 h post-mixing (**SL24h**) by a single trained observer. Recently received lesions were counted separately on three regions of the body: 1) anterior (i.e., head, neck, front legs, shoulders), 2) central (i.e., flanks and back), and 3) posterior (i.e., hind quarters and rear legs). One uninterrupted scratch was classed as a single lesion, regardless of length or severity. A lesion was considered as recent if it was vivid red in color or recently scabbed. The pre-mixing lesion count was subtracted from that taken 24 h post-mixing for each pig. This served to ensure that only those lesions that occurred as a result of mixing aggression were included in all analyses.

### Statistical analysis

Skin lesion showed considerably skewed distributions (Table 2) and thus, a log transformation was used to approach the normal distribution. The transformed values were used to estimate variance components. Similarly, although CRATE and IHAT responses were scored on an ordinal scale, the skewness and kurtosis of the data (Table 2) indicated that the traits followed an approximately normal distribution. Associations between predicted and predictor variables were tested using linear mixed models in *R* v. 4.1.2 (R Core Team., 2021). Predictors with a *P* < 0.05 were selected for inclusion in the variance component models. Genetic analyses were performed using DMU v6.5.2 (Madsen and Jensen, 2013) using the average information (DMU AI) restricted maximum likelihood (**REML**) algorithm. Each trait was analyzed using single-trait animal models. Models for CRATE and IHAT responses and skin lesions followed the general formula:

**Table 2.** Descriptive statistics for skin lesions^1^ 24 h and 5 wk post-mixing and individual behavioral responses of growing pigs isolated in a weigh crate^2^ or in a pen^3^ within 48 h post-mixing

		Original Scale						Transformed scale			
	*n*	Mean	SD	Min	Max	Skweness	Kurtosis	Mean	SD	Skweness	Kurtosis
*Skin lesions 24 h post-mixing*											
Anterior	2,013	17.9	14.34	1	92	1.4	2.6	1.1	0.43	−0.8	0.4
Central	2,013	15.9	13.30	1	82	1.5	2.9	1.0	0.46	−0.8	0.1
Posterior	2,012	9.7	8.19	1	52	1.6	3.2	0.8	0.41	−0.5	−0.4
*Skin lesions 5 wk post-mixing*											
Anterior	1,974	3.6	3.29	1	30	2.2	7.6	0.4	0.35	0.3	−1.0
Central	1,975	3.1	2.94	1	29	2.6	10.3	0.3	0.33	0.5	−0.6
Posterior	1,975	2.3	2.12	1	20	3.1	14.1	0.2	0.28	0.9	0.0
*Crate response*	1,844	3.2	0.83	2	5	0.33	−0.42	NA^4^	NA	NA	NA
*Individual human approach test*								NA	NA	NA	NA
Movement	2,014	3.0	0.72	1	6	−0.26	−0.02	NA	NA	NA	NA
Vocalization	2,014	1.8	0.78	1	5	0.53	−0.82	NA	NA	NA	NA
Vigilance	2,014	1.8	0.67	1	5	0.35	−0.48	NA	NA	NA	NA

^1^Lesions were counted separately on three regions of the body: 1) anterior (i.e., head, neck, front legs, shoulders), 2) central (i.e., flanks and back), and 3) posterior (i.e., hind quarters and rear legs). One uninterrupted scratch was classed as a single lesion, regardless of length or severity.

^2^Pigs remained isolated in a weigh crate for approximately 1 min and they were scored based on their restlessness on a 4-point scale where 1 = pig performed exploratory behavior including sniffing and rooting of the crate floor and walls; and 4 = pig performed serious, persistent attempts to escape by jumping over crate wall.

^3^After approximately 30 s after the pig entered a testing arena, a human observer walked toward the pig at a steady pace starting in the same corner of the pen each time and recorded the animal’s reaction to their approach. Three separate scores were given for each individual based on the severity of movement (score 0 = none to 3 = run), vocalizations (score 0 = none to 2 = loud grunts), and vigilance (score 0 = none to 2 = high).

^4^NA= Not applicable/ no transformation was applied to the data


$$\begin{array}{l} y=Xb+Za+Wc+\;e \end{array}$$

where:

*y* = vector of recorded traits

*b*, *a*, *c*, and *e* = vectors of the fixed effects, additive genetic effects, common environmental effects (i.e., pens where animals were mixed into), and the residual error, respectively. The fixed effect vector *b* contained genetic line, sex, and batch effects for all traits. Additionally, the order the animals were tested in was also included for CRATE and IHAT responses models. Body weight at mixing was fitted as a linear covariate for all traits.

*X*, *Z*, and *W* = Incidence matrices of fixed, additive genetic, and common environmental effects, respectively.

For the WTP traits, an animal model with the logit function for binary traits was used. Models followed the general formula:


$$\begin{array}{l} y=Xb+Za+e \end{array}$$

where:

*y* = vector of recorded traits

*b*, *a*, and *e* = vectors of the fixed effects, additive genetic effects, and the residual error, respectively. The fixed effect vector b contained the genetic line, sex, and batch effects for all traits.

*X* and *Z* = Incidence matrices of fixed, and additive genetic effects, respectively.

A seven-trait model was built for all linear variables. Genetic, phenotypic, and residual variances resulting from the single-trait animal models were used as starting values for the multi-trait model. Heritability, genetic and phenotypic correlation estimates were obtained by using an accompanying R program provided by DMU based on the notes “Calculation of Standard Errors of estimates of genetic and phenotypic parameters in DMU” by Jensen and Madsen (2002). Standard errors estimates were calculated from asymptotic standard errors of the corresponding variance components, which were obtained from the REML analyses using Taylor series approximations (Jensen and Madsen, 2002).

Multi-traits models including the WTP test traits failed to converge. Spearman correlations between estimated breeding values for WTP, CRATE response, IHAT traits, and skin lesions were calculated in R v. 4.1.2 (R Core Team, 2021) as a proxy for genetic correlations. Similarly, phenotypic correlations within these traits were estimated on the observed values using Spearman correlations in R v. 4.1.2 (R Core Team, 2021).

## Results

**Figure 2.**
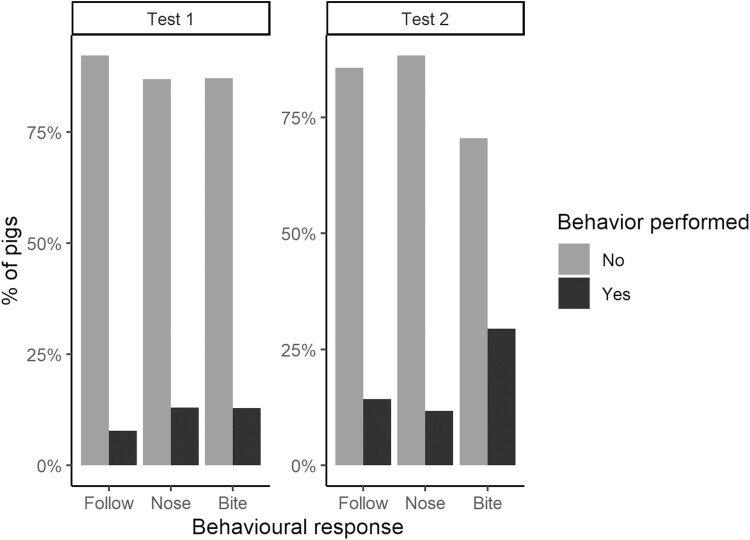
Percentage of growing pigs performing each behavior during the walk-the-pen test where pigs were tested for behavioral responses to the presence of a single human observer in their home pen at 6 ± 4.9 (Test 1) and 25 ± 15.9 (Test 2) days post-mixing. The observer walked around the pen and noted individuals that nosed (i.e., nosed or rooted at the observer’s boots or legs), followed (i.e., pig followed the observed around the pen) or bit (i.e., pig bit at the observer’s legs) the observer.

Descriptive statistics for skin lesions, CRATE, and IHAT responses are presented in Table 2. The proportion of pigs performing each behavior during the WTP test are shown in Figure 2.

### Heritabilities, common environmental effects, and phenotypic variance

Estimated heritabilities for skin lesion, CRATE, and IHAT responses were low to moderate (0.07 ± 0.02 to 0.29 ± 0.05), with the highest heritability estimated for speed of moving away from the approaching observer (Table 3). All heritabilities significantly differed from zero for these traits. Heritabilities for the WTP test traits were associated with high standard errors and were mainly non-significantly different from zero. Low but significant heritabilities were estimated for BITE T2 (0.11 ± 0.04) and NOSE T2 (0.09 ± 0.04; Table 4). Additive genetic variance ranged from 0.01 ± 0.05 to 0.36 ± 0.27 while phenotypic variance estimates were higher ranging from 0.44 ± 0.01 to 0.90 ± 0.03. For CRATE and IHAT response, pen effects accounted for little of the phenotypic variation and did not differ from zero. For all skin lesion traits, the phenotypic proportions of variances due to pen effects was similar and significantly differed from zero.

**Table 3.** Heritabilities (*h*^2^), additive (σ^2^_A_) and phenotypic variance (σ^2^_P_) and common environmental effects (*c*^2^) for skin lesions^1^ and behavioral responses of growing pigs to isolation in a weigh crate (i.e., CRATE response^2^), to a human approaching while isolated in an arena^3^ and to a human while walking in their home pen^4^. Standard errors are presented in parentheses.

Trait	*n*	*h* ^2^	σ^2^_A_	σ^2^_P_	*c* ^2^
*CRATE response*	1,844	0.21 (0.05)	0.14 (0.02)	0.67 (0.02)	0.01 (0.01)
*Individual human approach test*					
Movement	2,014	0.29 (0.05)	0.15 (0.03)	0.52 (0.02)	0.00 (0.01)
Vocalization	2,014	0.17 (0.04)	0.10 (0.02)	0.59 (0.02)	0.01 (0.01)
Vigilance	2,014	0.19 (0.04)	0.08 (0.02)	0.44 (0.01)	0.00 (0.001)
*Walk-the-pen test*					
Follow T1	2,023	0.26 (0.27)	0.36 (0.27)	N/A^5^	N/A
Follow T2	2,413	0.25 (0.16)	0.34 (0.16)	N/A	N/A
Nose T1	2,023	0.12 (0.17)	0.15 (0.17)	N/A	N/A
Nose T2	2,413	0.09 (0.04)	0.01 (0.05)	N/A	N/A
Bite T1	2,023	0.24 (0.19)	0.33 (0.20)	N/A	N/A
Bite T2	2,413	0.11 (0.04)	034 (0.12)	N/A	N/A
*Skin lesions 24 h post-mixing*					
Anterior	2,013	0.07 (0.02)	0.05 (0.02)	0.83 (0.29)	0.15 (0.02)
Central	2,013	0.10 (0.03)	0.09 (0.03)	0.90 (0.03)	0.14 (0.02)
Posterior	2,013	0.14 (0.03)	0.10 (0.02)	0.75 (0.03)	0.14 (0.02)

^1^Lesions were counted separately on three regions of the body: 1) anterior (i.e., head, neck, front legs, shoulders), 2) central (i.e., flanks and back), and 3) posterior (i.e., hind quarters and rear legs). One uninterrupted scratch was classed as a single lesion, regardless of length or severity.

^2^Pigs remained isolated in a weigh crate for approximately 1 min and they were scored based on their restlessness on a 4-point scale where 1 = pig performed exploratory behavior including sniffing and rooting of the crate floor and walls; and 4 = pig performed serious, persistent attempts to escape by jumping over crate wall.

^3^After approximately 30 s after the pig entered a testing arena, a human observer walked toward the pig at a steady pace starting in the same corner of the pen each time and recorded the animal’s reaction to their approach. Three separate scores were given for each individual based on the severity of movement (score 0 = none to 3 = run), vocalizations (score 0 = none to 2 = loud grunts), and vigilance (score 0 = none to 2 = high).

^4^Pigs were tested for behavioral responses to the presence of a single human observer while walking in their home pen at 6 ± 4.9 (T1) and 25 ± 15.9 (T2) days post-mixing. The observer walked around the pen and noted individuals that nosed (i.e. nosed or rooted at the observer’s boots or legs), followed (i.e., pig followed the observed around the pen) or bit (i.e., pig bit at the observer’s legs) the observer.

^5^Estimates are not available because a logistic model was fitted for these binary traits.

**Table 4.** Genetic (above the diagonal) and phenotypic (below the diagonal) correlations for skin lesions^1^ and for behavioral responses of growing pigs to isolation in a weigh crate (i.e. CRATE response^2^) and to a human approaching while isolated in an arena^3^. Standard errors are presented in parentheses

	Crate response	Movement	Vocalization	Vigilance	Anterior 24 h	Central 24 h	Posterior 24 h
Crate response		0.60 (0.11)	0.53 (0.14)	0.52 (0.15)	0.20 (0.17)	0.31 (0.15)	0.39 (0.15)
Movement	0.22 (0.02)		0.60 (0.11)	0.93 (0.06)	−0.03 (0.15)	−0.10 (0.14)	−0.07 (0.14)
Vocalization	0.32 (0.02)	0.41 (0.02)		0.72 (0.12)	0.07 (0.17)	0.08 (0.16)	0.04 (0.16)
Vigilance	0.11 (0.02)	0.44 (0.02)	0.25 (0.02)		0.03 (0.17)	−0.03 (0.15)	0.03 (0.15)
Anterior 24 h	−0.01 (0.02)	0.01 (0.02)	0.01 (0.02)	−0.01 (0.02)		0.91 (0.11)	0.79 (0.14)
Central 24 h	−0.01 (0.03)	0.01 (0.02)	0.02 (0.02)	0.01 (0.02)	0.63 (0.02)		0.91 (0.08)
Posterior 24 h	−0.02 (0.03)	−0.01 (0.02)	0.03 (0.02)	0.01 (0.02)	0.54 (0.02)	0.72 (0.02)	

^1^Lesions were counted separately on three regions of the body 24 h (SL24h) and 5 wk (SL5WK) post-mixing: 1) anterior (i.e., head, neck, front legs, shoulders), 2) central (i.e., flanks and back), and 3) posterior (i.e., hind quarters and rear legs). One uninterrupted scratch was classed as a single lesion, regardless of length or severity.

^2^Pigs remained isolated in a weigh crate for approximately 1 min and they were scored based on their restlessness on a 4-point scale, where 1 = pig performed exploratory behavior including sniffing and rooting of the crate floor and walls; and 4 = pig performed serious, persistent attempts to escape by jumping over crate wall.

^3^ After approximately 30 s after the pig entered a testing arena, a human observer walked toward the pig at a steady pace starting in the same corner of the pen each time and recorded the animal’s reaction to their approach. Three separate scores were given for each individual based on the severity of movement (score 0 = none to 3 = run), vocalizations (score 0 = none to 2 = loud grunts), and vigilance (score 0 = none to 2 = high).

### Genetic correlations

Genetic correlations (**r**_**g**_) between CRATE response, IHAT traits and skin lesions are presented in Table 4. Significant positive high r_g_ were observed between CRATE and IHAT responses (0.52 to 0.93), and within the various skin lesions traits (0.79 to 0.91), while significant positive low to high correlations between the estimated breeding values **(r**_**EBV**_) were estimated for the measures recorded within the WTP test (0.24 to 0.59). Correlations between the estimated breeding values of CRATE and IHAT test responses and WTP test traits were low, mostly negative (−0.21 to 0.05; Table 5) and did not significantly differ from zero except for r_EBV_ between CRATE and NOSE T2. Low significant positive r_EBV_ (0.06 to 0.24) were observed between skin lesions and the WTP test traits.

**Table 5.** Correlations between estimated breeding values for behavioral responses of growing pigs to a human while walking in their home pen^1^, to isolation in a weigh crate (i.e. CRATE response^2^) and to a human approaching while isolated in an arena^3^ and for skin lesions^4^. Standard errors are presented in parentheses

	Follow 1	Nose 1	Bite 1	Follow 2	Nose 2	Bite 2
Follow 1	1.00	0.42 (0.02)	0.53 (0.02)	0.48 (0.02)	0.38 (0.02)	0.49 (0.02)
Nose 1	0.42 (0.02)	1.00	0.48 (0.02)	0.48 (0.02)	0.24 (0.02)	0.59 (0.02)
Bite 1	0.53 (0.02)	0.48 (0.02)	1.00	0.50 (0.02)	0.27 (0.02)	0.50 (0.02)
Follow 2	0.48 (0.02)	0.48 (0.02)	0.50 (0.02)	1.00	0.36 (0.02)	0.55 (0.02)
Nose 2	0.38 (0.02)	0.24 (0.02)	0.27 (0.02)	0.36 (0.02)	1.00	0.39 (0.02)
Bite 2	0.49 (0.02)	0.59 (0.02)	0.50 (0.02)	0.55 (0.02)	0.39 (0.02)	1.00
Crate response	-0.09 (0.03)	-0.04 (0.03)	-0.06 (0.02)	-0.19 (0.02)	-0.10 (0.02)	-0.07 (0.02)
Movement	-0.02 (0.02)	-0.07 (0.02)	-0.03 (0.02)	-0.19 (0.02)	-0.05 (0.02)	-0.06 (0.02)
Vocalisation	0.05 (0.02)	-0.002 (0.02)	-0.05 (0.02)	-0.09 (0.02)	-0.07 (0.02)	-0.02 (0.02)
Vigilance	-0.20 (0.02)	-0.11 (0.02)	-0.21 (0.02)	-0.19 (0.02)	-0.21 (0.02)	-0.12 (0.02)
Anterior 24h	0.06 (0.02)	0.06 (0.02)	0.04 (0.02)	0.11 (0.02)	0.11 (0.02)	0.001 (0.02)
Central 24h	0.23 (0.02)	0.19 (0.02)	0.11 (0.02)	0.12 (0.02)	0.11 (0.02)	0.24 (0.02)
Posterior 24h	0.17 (0.02)	0.07 (0.02)	0.03 (0.02)	0.17 (0.02)	0.12 (0.02)	0.09 (0.02)

^1^ Pigs were tested for behavioral responses to the presence of a single human observer while walking in their home pen at 6 ± 4.9 (T1) and 25 ± 15.9 (T2) days post-mixing. The observer walked around the pen and noted individuals that nosed (i.e. nosed or rooted at the observer’s boots or legs), followed (i.e. pig followed the observed around the pen) or bit (i.e. pig bit at the observer’s legs) the observer.

^2^ Pigs remained isolated in a weigh crate for approximately 1 minute and they were scored based on their restlessness on a 4-point scale where 1 = pig performed exploratory behavior including sniffing and rooting of the crate floor and walls; and 4 = pig performed serious, persistent attempts to escape by jumping over crate wall.

^3^ After approximately 30 seconds after the pig entered a testing arena, a human observer walked toward the pig at a steady pace starting in the same corner of the pen each time and recorded the animal’s reaction to their approach. Three separate scores were given for each individual based on the severity of movement (score 0 = none to 3 = run), vocalizations (score 0 = none to 2 = loud grunts), and vigilance (score 0 = none to 2 = high).

^4^ Lesions were counted separately on three regions of the body 24 h (SL24h) post-mixing: i) anterior (i.e. head, neck, front legs, shoulders), ii) central (i.e. flanks and back), and iii) posterior (i.e. hind quarters and rear legs). One uninterrupted scratch was classed as a single lesion, regardless of length or severity.

### Phenotypic correlations

Phenotypic correlations between CRATE response, IHAT traits and skin lesions are presented in Table 4. Phenotypic correlations between the aforementioned traits and the WTP traits are presented in Table 6. Significant positive low to moderate phenotypic correlations were observed between CRATE and IHAT responses (0.11 to 0.44) and between the various WTP test traits (0.11 to 0.46), while significant positive low to high phenotypic correlations were estimated between the skin lesion traits (0.54 to 0.72). Phenotypic correlations between CRATE and IHAT responses and skin lesions were low and did not significantly differ from zero. Phenotypic correlations between CRATE and IHAT responses and WTP test traits were low and not significantly different from zero except for the correlations between VIGILANCE and BITE T1 (−0.11). Similarly, phenotypic correlations between skin lesions and the WTP test traits were low and did not differ from zero except for the correlations between anterior SL24h and NOSE during both tests.

**Table 6.** Phenotypic correlations for behavioral responses of growing pigs to a human while walking in their home pen^1^, to isolation in a weigh crate (i.e. CRATE response^2^) and to a human approaching while isolated in an arena^3^ and for skin lesions^4^. Standard errors are presented in parentheses

	Follow 1	Nose 1	Bite 1	Follow 2	Nose 2	Bite 2
Follow 1	1.00	0.14 (0.02)	0.46 (0.02)	0.27 (0.02)	0.11 (0.02)	0.20 (0.02)
Nose 1	0.14 (0.02)	1.00	0.14 (0.02)	0.19 (0.02)	0.11 (0.02)	0.22 (0.02)
Bite 1	0.46 (0.02)	0.14 (0.02)	1.00	0.23 (0.02)	0.13 (0.02)	0.27 (0.02)
Follow 2	0.27 (0.02)	0.19 (0.02)	0.23 (0.02)	1.00	0.18 (0.02)	0.41 (0.02)
Nose 2	0.11 (0.02)	0.11 (0.02)	0.13 (0.02)	0.18 (0.02)	1.00	0.24 (0.02)
Bite 2	0.20 (0.02)	0.22 (0.02)	0.27 (0.02)	0.41 (0.02)	0.24 (0.02)	1.00
Crate response	−0.03 (0.02)	−0.01 (0.02)	−0.02 (0.02)	−0.05 (0.02)	0.01 (0.02)	−0.04 (0.02)
Movement	−0.02 (0.02)	0.05 (0.02)	−0.05 (0.02)	−0.05 (0.02)	−0.01 (0.02)	−0.01 (0.02)
Vocalization	−0.03 (0.02)	0.02 (0.02)	−0.04 (0.02)	−0.02 (0.02)	0.02 (0.02)	−0.02 (0.02)
Vigilance	−0.07 (0.02)	0.01 (0.02)	−0.11 (0.02)	−0.03 (0.02)	−0.01 (0.02)	−0.03 (0.02)
Anterior 24 h	0.01 (0.02)	0.05 (0.02)	0.003 (0.02)	0.02 (0.02)	0.05 (0.02)	−0.002 (0.02)
Central 24 h	−0.003 (0.02)	−0.01 (0.02)	−0.03 (0.02)	0.01 (0.02)	0.07 (0.02)	−0.01 (0.02)
Posterior 24 h	0.01 (0.02)	−0.01 (0.02)	−0.04 (0.02)	0.02 (0.02)	0.04 (0.02)	−0.004 (0.02)

^1^Pigs were tested for behavioral responses to the presence of a single human observer while walking in their home pen at 6 ± 4.9 (T1) and 25 ± 15.9 (T2) days post-mixing. The observer walked around the pen and noted individuals that nosed (i.e., nosed or rooted at the observer’s boots or legs), followed (i.e., pig followed the observed around the pen) or bit (i.e., pig bit at the observer’s legs) the observer.

^2^Pigs remained isolated in a weigh crate for approximately 1 min and they were scored based on their restlessness on a 4-point scale where 1 = pig performed exploratory behavior including sniffing and rooting of the crate floor and walls; and 4 = pig performed serious, persistent attempts to escape by jumping over crate wall.

^3^After approximately 30 s after the pig entered a testing arena, a human observer walked toward the pig at a steady pace starting in the same corner of the pen each time and recorded the animal’s reaction to their approach. Three separate scores were given for each individual based on the severity of movement (score 0 = none to 3 = run), vocalizations (score 0 = none to 2 = loud grunts), and vigilance (score 0 = none to 2 = high).

^4^Lesions were counted separately on three regions of the body 24 h (SL24h) post-mixing: 1) anterior (i.e., head, neck, front legs, shoulders), 2) central (i.e., flanks and back), and 3) posterior (i.e., hind quarters and rear legs). One uninterrupted scratch was classed as a single lesion, regardless of length or severity.

## Discussion

### Heritabilities

Heritabilities for all studied traits, where significant, were in the range from low to moderate. The heritability for behavior while in the weighing crate was similar to that reported by D’Eath et al. (2009), Holl et al. (2010), and Rohrer et al. (2013) of 0.17 ± 0.03, 0.23, and 0.19 ± 0.03, respectively suggesting that the *h*^2^ of behavioral reactions to confinement in a weighing crate is consistent across a range of populations and environments. In the present study, the highest *h*^2^ was estimated for speed of moving away from the human observer during the IHAT, which was higher than that of 0.15 ± 0.02 reported by Jones et al. (2009). This test was less subjective and less prone to observer error as the scoring system was open to little interpretation (i.e., movement was zero, walk, trot, or run). Although measures were chosen to be as objective as possible, perceptions of behavior while in the weighing crate, and vocalizations and vigilance during a human approach, were more subjective, which may have resulted in greater variability over time in how the scale was used. For example, the behavior of any given animal may seem more or less extreme in comparison to the animal tested previously, influencing how the observer scored subsequent animals.

It is reported that *h*^2^ for fearfulness and/or boldness declines with age, possibly due to habituation to handling through repeated testing (Haskell et al., 2014). This in line with the decline in heritability estimates for BITE and NOSE observed in this study at approximately 4 wk post-mixing when compared with *h*^2^ estimates within 1 wk post-mixing. The WTP test reflects the conflicting motivations to explore the human and to withdraw from them. It is likely that the first and second WTP test differed in the extent to which they invoked these contrasting motivations. In the second WTP test the exploratory behavior measured may have been greater because fear suppressed approach during the first WTP test. The *h*^2^ of skin lesion traits observed in this study were similar to the lower range of those reported by Turner et al. (2009) and Wurtz et al. (2017) of 0.19 to 0.43 and 0.10 to 0.40; respectively. Our results suggest that skin lesions, and the associated aggressive behavior, could be reduced by means of genetic selection.

The proportion of the variance due to pen effects was very small for the behavioral traits relating to CRATE and IHAT responses. This is in contrast to skin lesions, where pen effects accounted for 14% to 15% of the observed variation. As physical aggression is the result of interactions between animals, it is reasonable that pen effects account for more of the variation in this behavior. During the CRATE and IHAT tests, pigs were tested individually and thus, it was unlikely that the behavior of each pig was affected by its pen mates. Furthermore, pen effects did not contribute to explain the variation in the WTP test. Indeed, when pen effect was included in the model, they failed to converge. This was surprising given that behavior of pen mates is likely to influence the behavior of a pig. For example, a shy pig might feel more confident approaching a human after observing a pen mate approaching. It is possible that within each pen the behavior of pen mates influenced the individual behavioral reactions observed; however, between pen responses did not differ sufficiently to account for the variation observed across the population.

### Correlations

While no phenotypic correlations were observed between behavioral traits and skin lesions in this study, positive low genetic correlations were observed between CRATE and central and posterior SL24h. This means that pigs that react more aversively while restrained in a weighing crate would also receive more central and posterior lesions when mixed into unfamiliar groups. There is evidence to suggest that posterior lesions at mixing are often inflicted when a defeated pig is retreating from a fight, and that lesions to this body region may indicate a subordinate position in the social hierarchy (Turner et al., 2006). As skin lesions and response to the crate were not phenotypically correlated, the genetic correlation indicates that the relationship between these traits was not simply a carry-over effect of mixing stress driving an increased stress response in the crate. The more persistent attempts to escape the weighing crate would suggest that pigs are experiencing more fear while restrained. Indeed, at the genetic level, pigs receiving higher scores while isolated in the weigh scale also grunted more, and ran away from and focused their attention on an approaching human while isolated in an arena in the IHAT.

Behavioral responses during the WTP test were correlated across time points at both the genetic and phenotypic level indicating the first and second WTP test traits shared the same genetic basis. Moreover, behavioral responses recorded during the WTP test were also highly correlated among them, suggesting that pigs biting the observer were also those that followed and nosed the observer. This implies a general increase in exploratory drive and/or a reduction in fearfulness in these animals. Correlations based on the estimated breeding values between reactions during the WTP test to a human observer and aggressive behavior were low suggesting that social aggression in pigs is not a good indicator of human directed exploration or aggression. For instance, while conducting the experiment, it became apparent that biting behavior in this population of growing pigs was not motivated by aggression. When pigs bit the observer, it appeared to be driven by curiosity and playfulness, rather than frustration or dominance, as vocalizations, aggressive biting and charging behaviors were absent which are reported as distinctive aggressive behavioral characteristics (Marchant Forde, 2002). However, this warrants further investigation. A limitation of this study was the inability to perform more detailed observations while conducting the WTP test that would have been more informative than simply recording binary responses. For example, some pigs immediately followed the observer around both laps of the pen, and persistently bit at the observer for the whole test period, while some hesitantly approached and eventually bit at the observer. These behaviors are probably indicative of different levels of fearfulness and/or boldness; however, both pigs would have simply been recorded as having displayed biting behavior. Moreover, this test was designed to be used as a practical on-farm measure of pig–human interactions and thus, it was of interest to develop a quick and accurate method of measuring these behaviors. For both the IHAT and the WTP tests, it would be preferable for more than one observer to record the behavior, and inter-observer reliability should be estimated. Additionally, due to the relatively low number of pigs interacting with the observer during the WTP test, more phenotyping (i.e., increased sample size and number of time points), a longer period of walking around the pen and the recording of the latency to approach the observer are needed for more accurate estimates for the studied traits.

Genetic correlations between CRATE and IHAT traits on the one hand, and the correlation based on the estimates breeding values for the WTP tests traits on the other, were low and mostly negative. Behavior while in isolation may be affected by the stress associated with the novelty of the environment (Lewis et al., 2008) or the stress of isolation. Therefore, behavior under these conditions is likely to differ from behavior while in the home pen with pen mates. In addition, the nature of the traits measured differed between the IHAT and the WTP tests. As every pig was explicitly tested during the IHAT, a reaction was forced from each individual as the human approached. In contrast, although the observer walked around the perimeter of the pen during the WTP, no pigs were singled out and the behavior ultimately measured was a pig’s willingness to approach and interact with the observer. In this situation, a pig that did not approach the observer may have done so out of fear or indifference, therefore a score of zero for the recorded traits is likely to have captured opposing reactionary behaviors.

There were several aspects of the experimental procedures used in the present study that may have affected the observed results. Ideally, CRATE and IHAT responses would be carried out in a completely novel environment by an unfamiliar handler. Both the weighing crate and isolation pen were familiar to the animals, as they had been weighed in the same crate and held in the same pens by farm staff 1 or 2 d prior to the tests. Testing pigs within the same time point is also not ideal, as their perception of the crate could carry over and affect their response to the IHAT, meaning that the tests were not independent. In addition, these pigs were already familiar with the observer carrying out the experiments, as the same observer had previously recorded skin lesions, moved the animals to and from the home pen, as well as moved them into the weighing crate. How aversive the pigs found these events may have affected their behavior in these tests.

In conclusion, under the conditions of this study, heritability estimates for all studied traits were in a range that suggests they could be suitable as a method of phenotyping aggression and fear/boldness for selection purposes in pigs. Results indicate that the genetic determination of the behavioral response to a human walking in the home pen declines with age. The decreased heritability estimates for the walk-the-pen test traits were likely associated with pigs becoming habituated to routine handling and/or repeated testing. Moreover, there was evidence of genetic associations between aggression and fear in pigs as those with higher central and posterior skin lesion counts 24 h post-mixing (i.e., likely to be subordinate pigs) tended to display more distress while in the weigh crate and were less likely to willingly approach a human in the IHAT. Conversely, pigs with a high number of lesions to the anterior part of the body 24 h post-mixing, which are typically the most numerous and received primarily during reciprocated attack, also showed an aversive reaction to being in the crate, but these animals were more willing to explore a human in their home pen in the WTP test. Exerting selection pressure to reduce the accumulation of lesions is therefore likely to make pigs more relaxed in a crate environment, but to alter the engagement with humans in other contexts that depends on the location of the lesions under selection. Future studies could consider using precision livestock farming technologies to assess animal-human interactions in a more detailed and objective manner and thus remove some of the possible confounding factors associated with the recording of behavioral observations. Finally, the findings reported in this study could have practical implications for the pig industry as they suggest that pigs selected for reduced aggression could be easier to handle while performing certain routine farm procedures such as weighing. Additionally, as less fearful animals have higher growth rates, higher carcass quality characteristics and better immune function (Kadel et al., 2006; Burdick et al., 2011) this could also impact performance traits and ultimately farm profitability; however, this warrants further investigation.

## Abbreviations

BITE: pig bit at the observer’s legs
CRATE: individual behavioral responses to isolation in a weighing crate test
FOLLOW: pig followed the observed around the pen
IHAT: individual human approach test in an arena
MOVEMENT: speed of movement away from the approaching observer
NOSE: pig nosed or rooted at the observer’s boots or legs
PIC: Pig Improvement Company
r_g_: genetic correlations
r_EBV_: correlations on the breeding values
REML: restricted maximum likelihood
SL: skin lesions
SL24h: skin lesions recorded 24 h post mixing
T1: first testing period for the walk-the-pen test
T2: second testing period the walk-the-pen test
VIGILANCE: pigs glancing/focusing on the approaching observer
VOCALISE: pigs vocalizing (i.e., grunts/squeals) while the observer approaches them
WTP: walk-the-pen test

## References

[CIT0001] Azarpajouh, S., J. A. Calderón Díaz, S. Bueso Quan, and H. Taheri. 2021. Farm 4.0: innovative smart dairy technologies and their applications as tools for welfare assessment in dairy cattle. CABI Rev. doi:10.1079/PAVSNNR202116045

[CIT0002] Bolhuis, J. E., W. G. P. Schouten, J. W. Schrama, and V. M. Wiegant. 2005a. Behavioural development of pigs with different coping characteristics in barren and substrate-enriched housing conditions. Appl. Anim. Behav. Sci. 93:213–228. doi:10.1016/j.applanim.2005.01.006

[CIT0003] Bolhuis, J. E., W. G. P. Schouten, J. W. Schrama, and V. M. Wiegant. 2005b. Individual coping characteristics, aggressiveness and fighting strategies in pigs. Anim. Behav. 69:1085–1091. doi:10.1016/j.anbehav.2004.09.013

[CIT0004] von Borstel, U. K., B. Tönepöhl, A. K. Appel, B. Voß, H. Brandt, S. Naderi, and M. Gauly. 2019. Suitability of traits related to aggression and handleability for integration into pig breeding programmes: Genetic parameters and comparison between Gaussian and binary trait specifications. PLoS One 13. doi:10.1371/journal.pone.0204211 PMC6310294 30592711

[CIT0005] Burdick, N. C., R. D. Randel, J. A. Carroll, and T. H. Welsh. 2011. Interactions between temperament, stress, and immune function in Cattle. Int J Zool. 2011:1–9. doi:10.1155/2011/373197

[CIT0006] D’Eath, R. B., R. Roehe, S. P. Turner, S. H. Ison, M. Farish, M. C. Jack, and A. B. Lawrence. 2009. Genetics of animal temperament: Aggressive behaviour at mixing is genetically associated with the response to handling in pigs. Animal 3:1544–1554. doi:10.1017/S1751731109990528 22444987

[CIT0007] Fels, M., J. Hartung, and S. Hoy. 2014. Social hierarchy formation in piglets mixed in different group compositions after weaning. Appl. Anim. Behav. Sci. 152:17–22. doi:10.1016/j.applanim.2014.01.003

[CIT0008] Forkman, B., A. Boissy, M. C. Meunier-Salaün, E. Canali, and R. B. Jones. 2007. A critical review of fear tests used on cattle, pigs, sheep, poultry and horses. Physiol. Behav. 92:340–374. doi:10.1016/j.physbeh.2007.03.016 18046784

[CIT0009] Haskell, M. J., G. Simm, and S. P. Turner. 2014. Genetic selection for temperament traits in dairy and beef cattle. Front. Genet. 5:368. doi:10.3389/fgene.2014.00368 25374582 PMC4204639

[CIT0010] Holl, J. W., G. A. Rohrer, and T. M. Brown-Brandl. 2010. Estimates of genetic parameters among scale activity scores, growth, and fatness in pigs. J. Anim. Sci. 88:455–459. doi:10.2527/jas.2008-1559 19820051

[CIT0011] Jensen, J., and P. Madsen. 2002. Calculation of standard errors of estimation of genetic and phenotypic parameters in DMU. Danish Institute of Agricultural Sciences, Research Centre Foulum.

[CIT0012] Jones, R. M., S. Hermesch, and R. E. Crump. 2009. Evaluation of pig flight time, average daily gain and backfat using random effect models including grower group. Proc. Assoc. Advmt. Anim. Breed. Genet. 18:199–202.

[CIT0013] Kadel, M. J., D. J. Johnston, H. M. Burrow, H. -U. Graser, and D. M. Ferguson. 2006. Genetics of flight time and other measures of temperament and their value as selection criteria for improving meat quality traits in tropically adapted breeds of beef cattle. Aust. J. Agric. Res. 57:1029. doi:10.1071/ar05082

[CIT0014] Lewis, C. R. G., L. E. Hulbert, and J. J. McGlone. 2008. Novelty causes elevated heart rate and immune changes in pigs exposed to handling, alleys, and ramps. Livest Sci 116:338–341. doi:10.1016/j.livsci.2008.02.014

[CIT0015] Madsen, P., and J. Jensen. 2013. A user’s guide to DMU. A package for analysing multivariate mixed models. Version 6, release 5.2. Tjele, Denmark: Center for Quantitative Genetics and Genomics, University of Aarhus.

[CIT0016] Marchant Forde, J. N. 2002. Piglet-and stockperson-directed sow aggression after farrowing and the relationship with a pre-farrowing, human approach test. Appl. Anim. Behav. Sci. 75:115–132. doi:10.1016/s0168-1591(01)00170-8

[CIT0017] Melotti, L., M. Oostindjer, J. E. Bolhuis, S. Held, and M. Mendl. 2011. Coping personality type and environmental enrichment affect aggression at weaning in pigs. Appl. Anim. Behav. Sci. 133:144–153. doi:10.1016/j.applanim.2011.05.018

[CIT0018] Norris, D., J. W. Ngambi, M. Mabelebele, O. J. Alabi, and K. Benyi. 2014. Genetic selection for docility: A review. J Anim Plant Sci. 24:374–379.

[CIT0019] R Core Team. 2021. R: A language and environment for statistical computing. Vienna, Austria: R Foundation for Statistical Computing. https://www.R-project.org/

[CIT0020] Rodrigues da Costa, M., E. García Manzanilla, A. Diana, N. van Staaveren, A. Torres-Pitarch, L. A. Boyle, and J. A. Calderón Díaz. 2021. Identifying challenges to manage body weight variation in pig farms implementing all-in-all-out management practices and their possible implications for animal health: a case study. Porcine Health Manag. 7. doi:10.1186/s40813-021-00190-6 PMC7798213 33431068

[CIT0021] Rohrer, G. A., T. Brown-Brandl, L. A. Rempel, J. F. Schneider, and J. Holl. 2013. Genetic analysis of behavior traits in swine production. Livest Sci 157:28–37. doi:10.1016/j.livsci.2013.07.002

[CIT0022] Ruis, M. A. W., J. H. A. te Brake, J. A. van de Burgwal, I. C. de Jong, H. J. Blokhuis, and J. M. Koolhaas. 2000. Personalities in female domesticated pigs: behavioural and physiological indications. Appl. Anim. Behav. Sci. 66:31–47. doi:10.1016/S0168-1591(99)00070-2

[CIT0023] Scheffler, K., E. Stamer, I. Traulsen, and J. Krieter. 2014. Genetic analysis of the individual pig behaviour in backtests and human approach tests. Appl. Anim. Behav. Sci. 160:38–45. doi:10.1016/j.applanim.2014.08.010

[CIT0024] Turner, S. P., M. J. Farnworth, I. M. S. White, S. Brotherstone, M. Mendl, P. Knap, P. Penny, and A. B. Lawrence. 2006. The accumulation of skin lesions and their use as a predictor of individual aggressiveness in pigs. Appl. Anim. Behav. Sci. 96:245–259. doi:10.1016/j.applanim.2005.06.009

[CIT0025] Turner, S. P., R. Roehe, R. B. D’Eath, S. H. Ison, M. Farish, M. C. Jack, N. Lundeheim, L. Rydhmer, and A. B. Lawrence. 2009. Genetic validation of postmixing skin injuries in pigs as an indicator of aggressiveness and the relationship with injuries under more stable social conditions. J. Anim. Sci. 87:3076–3082. doi:10.2527/jas.2008-1558 19574573

[CIT0026] Wurtz, K. E., J. M. Siegford, R. O. Bates, C. W. Ernst, and J. P. Steibel. 2017. Estimation of genetic parameters for lesion scores and growth traits in group-housed pigs. J. Anim. Sci. 95:4310–4317. doi:10.2527/jas2017.1757 29108070

